# Nanotechnological approaches for counteracting multidrug resistance in cancer

**DOI:** 10.20517/cdr.2020.47

**Published:** 2020-10-12

**Authors:** Chiara Martinelli, Marco Biglietti

**Affiliations:** ^1^Independent Researcher, Como 22100, Italy.; ^2^Independent Researcher, Milan 20148, Italy.

**Keywords:** Multidrug resistance, cancer, nanotechnology, nanomedicine, nanocarriers, targeted delivery

## Abstract

Every year, cancer accounts for a vast portion of deaths worldwide. Established clinical protocols are based on chemotherapy, which, however, is not tumor-selective and produces a series of unbearable side effects in healthy tissues. As a consequence, multidrug resistance (MDR) can arise causing metastatic progression and disease relapse. Combination therapy has demonstrated limited responses in the treatment of MDR, mainly due to the different pharmacokinetic properties of administered drugs and to tumor heterogeneity, challenges that still need to be solved in a significant percentage of cancer patients. In this perspective, we briefly discuss the most relevant MDR mechanisms leading to therapy failure and we report the most advanced strategies adopted in the nanomedicine field for the design and evaluation of *ad hoc* nanocarriers. We present some emerging classes of nanocarriers developed to reverse MDR and discuss recent progress evidencing their limits and promises.

## Introduction

**Figure 1:**
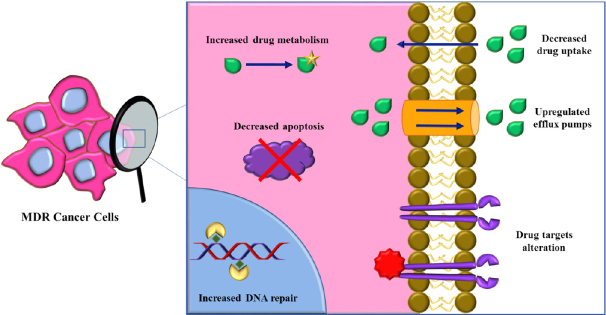
Scheme of the most relevant mechanisms involved in MDR onset in cancer. The upregulation of efflux pumps is responsible for excessive drug efflux with consequently decreased drug uptake. Drugs can be metabolized by upregulated enzymes and drug targets can be modified. Apoptosis is often deregulated and there can be an increased DNA damage repair. MDR: multidrug resistance

Cancer is one of the prominent causes of death worldwide with an estimate of an increase of up to approximately 13.2 million cancer-related deaths a year by 2030^[1]^. Conventional cancer treatments rely on chemotherapy, that unfortunately does not display sufficient selectivity for tumor cells and very often causes many severe adverse effects in already debilitated patients. Notably, drug resistance can occur after or even during treatment, and cancer cells can eventually become concurrently resistant not only to the administered therapeutic agent but also to different kinds of unrelated drugs, leading to multidrug resistance (MDR)^[2]^ and consequent therapy failure. MDR is responsible for the vast majority of tumor metastasis and relapse^[3]^ and accounts for over 90% of chemotherapy failures in patients with metastatic cancer^[4]^. Many mechanisms involved in this phenomenon have been identified, such as (1) alterations in the apoptotic cascade and DNA damage repair; (2) modifications in the molecules targeted by drugs; (3) drug detoxification by upregulated enzymes; and (4) overexpression of ATP-binding cassette (ABC) pumps [e.g., P-glycoprotein (P-gp)] responsible for excessive drug efflux^[2,5]^ [Figure 1]. Additionally, modifications in the tumor microenvironment, such as abnormal vasculature, localized hypoxia, and low pH, contribute to limited drug penetration^[6,7]^. Different approaches have been evaluated for avoiding chemotherapy (i.e., surgery and radiotherapy) or circumventing MDR (i.e., small molecule inhibitors, chemo-sensitizers, gene therapy). However, to achieve effective doses, these strategies have often demonstrated to be highly dangerous for patient’s survival. Drug combinations have been used, simultaneously targeting different pathways: chemotherapeutic agents have been combined with P-glycoprotein inhibitors and tyrosine kinase inhibitors or proapoptotic agents have been employed for enhancing cytotoxicity^[8,9]^. Nevertheless, the outcome of these treatments may be unpredictable due to the different pharmacokinetic properties of each molecule and specific doses required.

The advent of nanomedicine has prompted the clinicians with new tools for counteracting MDR, thanks to the rational design of nanocarriers able to deliver multiple treating agents to cancer cells, with prolonged systemic circulation, exploiting the leaky vasculature and abnormal lymphatic drainage [enhanced permeability and retention (EPR)] of solid tumors^[10-12]^ and presenting efficient drug release, thus minimizing possible adverse side effects.

A plethora of nanomaterials has been explored, ranging from inorganic (e.g., iron oxide nanoparticles^[13]^, gold nanoparticles^[14-16]^, quantum dots^[17]^, mesoporous silica nanoparticles^[18,19]^, carbon nanotubes^[20,21]^) to organic ones (e.g., liposomes^[22,23]^, polymeric nanoparticles^[24]^, micelles^[25]^, dendrimers^[26]^). Nevertheless, nanocarriers working merely as passive drug delivery systems present some drawbacks partially limiting their wide application for cancer therapy^[27]^. For instance, nanoparticles can release their cargo during circulation and before reaching their target cells. Therefore, they need to be functionalized with targeting ligands able to univocally recognize and bind tumor cells^[28]^, avoiding damage to healthy ones^[29]^. Active targeting allows drug endocytosis, thus bypassing ABC transporters responsible for cytotoxic drug efflux once they are released into the cytoplasm^[30]^. Furthermore, stimuli-responsive nanomedicines have been developed, able to perform active delivery, and triggered the release of the drug, upon reaching their target tumor^[31,32]^. In the last decade, many studies have demonstrated the great potential of nanocarriers to overcome cancer-related MDR^[33,34]^.

This perspective focuses on describing different kinds of inorganic and organic nanomaterials and the most innovative approaches employed for counteracting MDR. The most significant researches in preclinical context are reported, evidencing possible limits, and underlining the promises for their future clinical application.

## Inorganic nanocarriers

**Figure 2:**
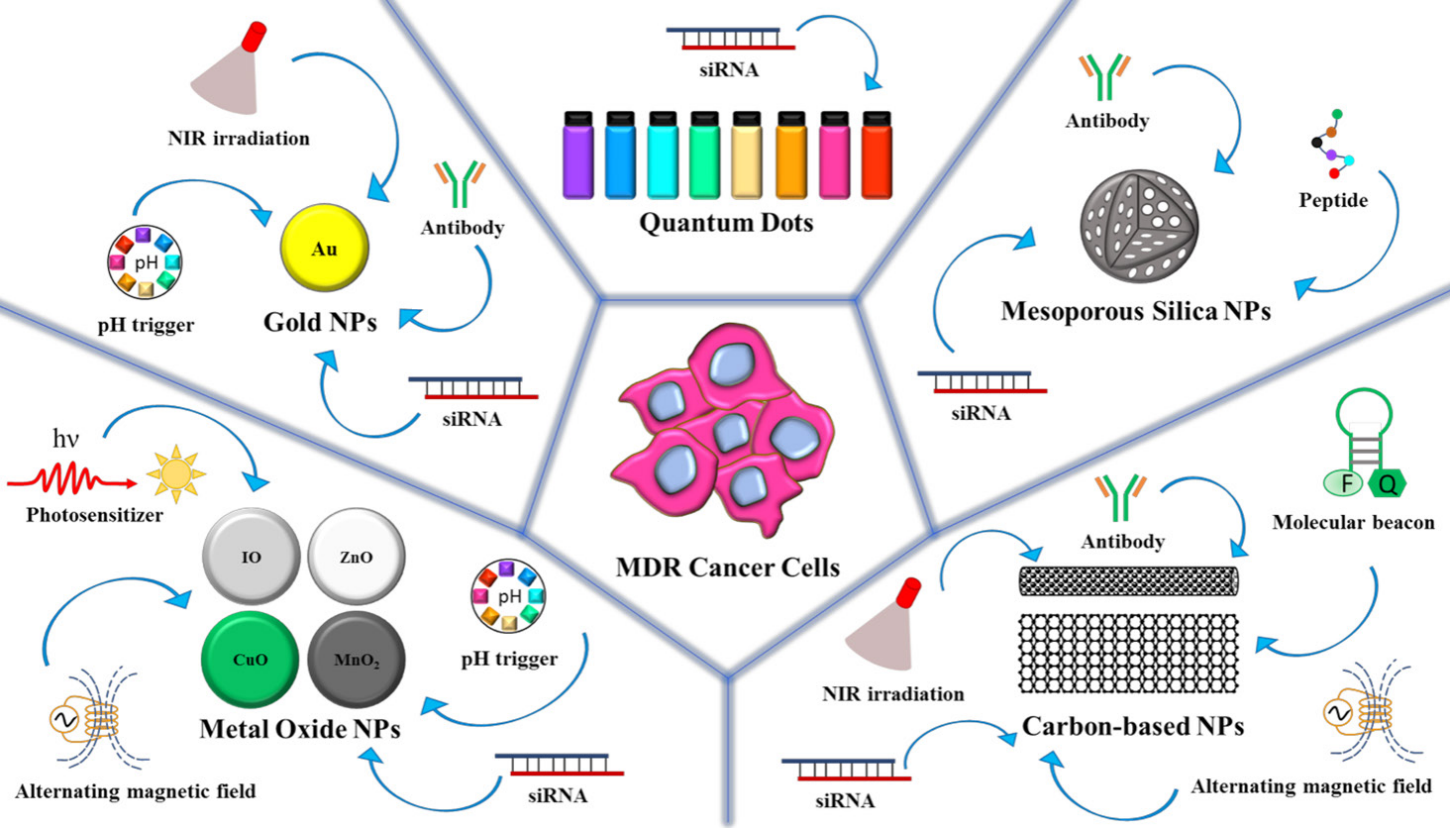
Scheme of the main inorganic nanoparticles investigated for counteracting MDR in cancer. From the left to the right side, metal oxide nanoparticles (NPs), gold nanoparticles, quantum dots, mesoporous silica nanoparticles, and carbon-based nanoparticles are depicted. Possible functionalization with targeting ligands and external stimuli exploited for triggered drug release are reported. IO: iron oxide; MDR: multidrug resistance; NIR: near-infrared

Several inorganic nanoparticles have been exploited for drug delivery, presenting a plethora of advantages respect to conventional anticancer approaches [Figure 2]. For instance, high surface-to-volume ratio, tunable properties in response to external stimuli (e.g., light, temperature, magnetic field), possibility to achieve the desired size and shape, and to be easily surface modified with targeting ligands and/or fluorophores for *in vivo* MDR cancer imaging.

### Iron oxide and metal oxide nanoparticles

Iron oxide nanoparticles have been widely employed in the biomedical field, thanks to their biocompatibility. They have been applied as contrast agents (superparamagnetic iron oxide nanoparticles, SPIONs), as biosensors and for hyperthermia treatments^[35,36]^. Moreover, they have been exploited as efficient drug delivery vehicles able to release their cargo mainly upon endocytosis, without being intercepted by the pump transporters present on MDR cancer cells. For instance, iron oxide nanoparticles efficiently delivered doxorubicin in MDR HeLa cells, showing increased uptake and apoptosis induction^[37]^. In an interesting work, doxorubicin has been covalently bound to polyethyleneimine (PEI) *via* a pH-sensitive hydrazone linkage located on the surface of iron oxide nanoparticles. Upon nanoparticles administration, endosomal escape, and subsequent release of the drug in the cytoplasm of resistant rat glioma cells have been obtained, showing higher cellular retention respect to free doxorubicin^[38]^. A nanotheragnostic platform has been developed based on pH-sensitive magnetic nanoparticles prone to disassemble in the acidic tumor environment, giving rise to the possibility to be used as contrast agents for diagnosis and, upon loading with photosensitizers generating singlet oxygen, used to kill cancer resistant cells for photodynamic therapy^[39]^. As mentioned before, the administration of combination therapy, composed of inhibitors and chemotherapeutics, encounters many difficulties in overcoming MDR, due to the different pharmacokinetic properties of these kinds of agents^[40]^. Notably, nanocarriers can easily perform this duty, thanks to the fact that they can be loaded with multiple molecules and simultaneously deliver them to the diseased tissue^[41]^. In a pioneering work, iron oxide nanoparticles carrying 5-bromotetrandrine efficiently increased cytotoxicity in MDR cell lines and favored daunorubicin accumulation downregulating *mdr1* gene and P-gp expression, thus contributing to MDR reversal^[42]^. In an interesting study, iron oxide nanoparticles co-loaded with daunorubicin and wogonin were efficiently uptaken by cells and localized in endosomes, displaying a high degree of induced apoptosis and reduction of transcription of MDR1 mRNA and expression of P-gp in a leukemic cell line^[43]^. Hyperthermia, a technique developed for inducing cancer cell apoptosis by means of magnetic nanoparticles that generate heat when subjected to an alternating magnetic field^[44]^, has demonstrated to be efficient also for treating MDR cancers and, more specifically, for triggering drug release^[45]^. Iron oxide magnetic nanoparticles have been developed able to carry daunorubicin and 5-bromotetrandrine. They were administered in presence of an alternating magnetic field in an *in vivo* model of leukemia. Interestingly, P-gp and B-cell lymphoma 2 (Bcl-2) expression decreased, while Bcl-2 associated X and Caspase-3 expression increased, contributing to multidrug resistance reversal^[46]^. More recently, silk fibroin iron oxide nanoparticles carrying doxorubicin showed intracellular accumulation and cytotoxicity upon external magnetic field application in MDR breast cancer cells^[47]^. Iron oxide nanoparticles have been also engineered to directly impact MDR mechanisms and demonstrated efficient in carrying siRNAs for reducing the expression of P-gp, multidrug resistance-associated protein 1 (MRP1), and Bcl-2, protecting them from RNAse degradation and enhancing cell permeability and endosomal trapping^[48]^.

Altogether, these results demonstrate that iron oxide nanoparticles can work efficiently in overcoming MDR, even though further structural advances will be needed for obtaining the best carrier delivering a sufficient amount of drug. Moreover, regarding hyperthermia treatments, it has to be considered that the applied external magnetic field needs to be finely tuned in terms of frequency and exposure time, depending on each specific nanosystem in use.

Other metal oxide-based nanoparticles have shown MDR reversal properties. For instance, copper oxide and zinc oxide nanomaterials demonstrated to act as chemosensitizers inhibiting MDR transport mechanisms *in vivo*^[49]^. MnO_2_ nanosheets modified with polyethylene glycol (PEG) have been created for the fast release of doxorubicin in MDR cells. This modification made the nanomaterial very stable in physiological conditions and prone to break in the acidic intracellular environment. Thanks to their size, they also circumvented P-gp pumps preserving the drug inside the cells, while Mn^2+^ facilitated magnetic resonance imaging (MRI) of the tumor^[50]^.

### Gold nanoparticles

Similarly to iron oxide, gold has been exploited to develop highly biocompatible nanocarriers, very stable, and presenting good permeability in living cells. Notably, gold nanoparticles can be used for imaging applications, thanks to their electron density properties^[51,52]^. This kind of nanomaterial can be easily functionalized for carrying diverse cargoes in tumor cells^[53]^. Gold-doxorubicin nanoconjugates have been designed able to be efficiently internalized and release drugs in MDR cells, enhancing their cytotoxicity and overcoming MDR^[54]^. Gold nanoparticles functionalized with doxorubicin *via* an acid-labile linker prompted drug release in acidic compartments upon endocytosis and enhanced drug cytotoxicity inducing apoptosis in MDR breast cancer cells^[55]^. More elaborated systems, such as multilamellar gold niosomes, have been designed carrying the hydrophobic drug thymoquinone and, on the surface, siRNAs directed against Akt, a serine/threonine kinase responsible for regulating cell survival, insulin signaling, angiogenesis, and tumor formation. Its expression was reduced and apoptosis resulted enhanced in MDR breast cancer cells^[56]^. Another interesting application of engineered gold nanoparticles is photothermal therapy (PTT), which exploits the conversion of absorbed near-infrared (NIR) light to heat, for specifically destroying cancer cells^[57]^. Its successful application in the preclinical context has been reported^[58]^. For instance, doxorubicin-loaded gold nanoparticles functionalized with anti-death receptor-4 monoclonal antibody were employed for delivering high amounts of drug and concurrently reducing P-gp expression by PTT in MDR tumor xenografts^[59]^.

The reported researches demonstrate the high value of gold nanoparticles as delivery systems for MDR reversal. However, it is important to consider that synthesizing peculiar shapes implies the use of stabilizing agents that can reveal toxic for cells and/or that surface functionalization can make nanoparticles unstable. Therefore, biocompatible molecules, such as PEG and phospholipids, are required in order to render gold nanoparticles more stable, a quality essential for biomedical applications^[60]^.

### Quantum dots

Quantum dots (QDs) are a class of nanomaterials that can be employed as nanotheragnostic tools performing simultaneously imaging and therapeutic functions. Recently, graphene quantum dots have demonstrated to be able to downregulate P-gp and MRP1 by interacting with the C-rich regions of their promoters and efficiently reversing doxorubicin resistance in MCF-7/ADR cells^[61]^. CdSe/ZnS-MPA and CdSe/ZnS-GSH quantum dots have been evaluated for their ability to interact with P-gp, showing that they efficiently downregulated its expression in A549 cells, while upregulated miR-34b and miR-185, contributing to reveal their important modulatory function and possible future use as targets for cancer therapy^[62]^. Interestingly, they present unique optical and chemical features and have been engineered as drug delivery vehicles able to interfere with MDR mechanisms thanks to surface modification^[63,64]^. A remarkable study demonstrated that CdSe/ZnSe QDs were able to deliver doxorubicin and siRNAs targeting P-gp, reversing MDR in HeLa cells. The drug was efficiently released, bypassing P-gp mediated efflux, and siRNAs carried out their function. Moreover, apoptosis was induced and quantum dots allowed to image their localization by real-time imaging^[65]^.

Although very promising, further studies are currently required to better evaluate possible adverse effects linked to the intracellular release of heavy metal ions, to the production of reactive oxygen species (ROS) and QDs long-term toxicity in living organisms^[66]^.

### Mesoporous silica nanoparticles

Mesoporous silica nanoparticles (MSNs) present peculiar properties such as high pore volume and surface area resulting in high drug loading, tunable pore structure, and the possibility to be functionalized for targeted delivery^[67]^. For instance, they have been conjugated with monoclonal antibodies, peptides, nucleic acids and it has been shown that they are mainly internalized by micropinocytosis^[68]^. MSNs have been widely employed for drug loading aimed at MDR reversal mediated by passive or active targeting, thanks to the capacity to carry their cargo inside cells protecting it from the abnormal molecular mechanisms of MDR cancer cells^[69]^. Upon loading of doxorubicin in large pore MSNs, the drug was quickly released at high levels, inducing MDR reversal mediated by downregulation of P-gp^[70]^. Rod-shaped MSNs carrying doxorubicin were efficiently uptaken by MDR breast cancer cells^[71]^, while co-delivery of paclitaxel and tetrandrine were released in a pH-dependent manner completely overcoming MDR in breast cancer cells, with important clinical implications^[72]^. Arsenic trioxide (ATO), a DNA damage repair inhibitor drug used for treating acute promyelocytic leukemia, presents poor bioavailability and several side effects. Silica nanoparticles carrying simultaneously ATO and doxorubicin have demonstrated successful in killing MDR cells^[73]^. Many mechanisms are involved in the reversal of MDR mediated by MSNs. In a study, MSNs loaded with nuclear-targeted doxorubicin were administered in breast cancer MDR cells, and apoptosis was induced. MDR related gene expression resulted altered and DNA repair pathways impaired, with disruption of the p53 dependent tumor suppressor cascade^[74,75]^.

MSNs have been also employed for combined delivery of chemosensitizers and chemotherapeutic molecules^[76]^ and, engineered by surface modification, as carriers for MDR specific siRNAs^[77]^. In an interesting study, multifunctional MSNs, carrying doxorubicin and siRNAs targeting P-gp, efficiently delivered them in an MDR breast cancer xenograft model, displaying enhanced permeability and retention effect and synergistic inhibition of tumor growth with significant P-gp knockdown^[78]^. MSNs loaded with doxorubicin and functionalized with TAT peptide were composed of multiple layers self-assembled *via* electrostatic interactions. Vascular endothelial growth factor siRNAs were bound on the outer layer *via* disulfide bonds. Thanks to this strategy, siRNAs were released in the cytoplasm while doxorubicin delivered to the nucleus^[79]^.

Regarding this kind of nanomaterial, the most relevant drawbacks contributing to slowing down its clinical usage remain the difficulties related to large-scale synthesis and production and to their biodegradation rates, that continue to be still unclear and need further investigations^[80,81]^.

### Carbon-based nanocarriers

Carbon-based nanocarriers have demonstrated to be effective for biomedical applications due to their high surface-to-volume ratio, structural rigidity, thermal conductivity, and the possibility to be surface modified. In particular, carbon nanotubes (CNTs) have been widely employed as agents for cancer theragnostics^[82]^. Interestingly, among the several applications, they can be used as fluorescent imaging tools in the NIR range^[83]^ and as Raman probes for their scattering properties^[84]^. Carbon nanotubes can be subdivided into single-walled (SW) and multi-walled (MW), both of which modifiable with a plethora of different molecules^[85]^. Modified SWCNTs can easily work as nanocarriers penetrating every kind of cell, included MDR cancer cells^[86]^. Carbon nanotubes have been fabricated and loaded with paclitaxel and C6-ceramide. Upon administration to MDR pancreatic cancer cells, they caused high levels of apoptosis after heat generation by magnetic field induction^[87]^. Interestingly, SWCNTs covered by cholinic acid-derivatized hyaluronic acid (CAHA), efficiently delivered chemotherapeutic agents in MDR OVCAR8/ADR cells^[88]^. MWCNTs have been developed delivering *N*-desmethyl tamoxifen and quercetin (a P-gp inhibitor). In acidic conditions, tamoxifen was efficiently released in MDR MDA-MB-231 cells and *in vivo*^[89]^. Interestingly, hollow carbon nanospheres combined with NIR irradiation were capable of generating free radicals while releasing doxorubicin and have revealed efficient in MDR reversal^[90]^. In another study, MWCNTs have been functionalized with an anti-Pgp antibody and demonstrated to be able to induce high phototoxicity upon light irradiation in spheroids constituted by MDR cancer cells^[91]^.

Other widely employed carbon-based nanocarriers are graphene, a 2D crystal derived from graphite with intrinsic NIR fluorescence probes, and graphene oxide (GO), presenting absorbance properties in the NIR region, thus ideal for photoacoustic imaging^[82,92]^. Thanks to their planar structure, they can be easily loaded with several kinds of molecules interfering with MDR mechanisms. For instance, graphene has been exploited for delivering siRNAs together with anticancer drugs for targeting MDR cancers. GO carrying adriamycin and miR-21-targeted siRNAs was successfully administered to MDR cells^[93]^, while GO functionalized with chemotherapeutic agents has been used to treat MDR cancer also in combination with PTT^[94,95]^. Graphene oxide has been loaded with doxorubicin and irinotecan and stabilized by poloxamer 188. Upon NIR laser irradiation, generated heat was able to induce specific activation of apoptotic pathways and cytotoxicity in MDA-MB-231 resistant breast cancer cells^[96]^. Recently, graphene oxide modified with doxorubicin and two molecular beacons (MBs) was promptly uptaken by MDR breast cancer cells. In an acidic environment, doxorubicin was released and the two MBs bound their target sequences, effectively silencing MDR1 and ETS1 mRNAs and inducing P-gp inhibition *in vitro* and *in vivo*^[97]^.

Although very promising, carbon-based nanocarriers display an intrinsic tendency to aggregation, especially in physiological conditions, therefore *ad hoc* structural modifications are extremely important before their clinical application. For instance, it has to be considered that surface functionalization of graphene can induce significant variations in its chemical and physical properties^[98]^.

## Organic nanocarriers

**Figure 3:**
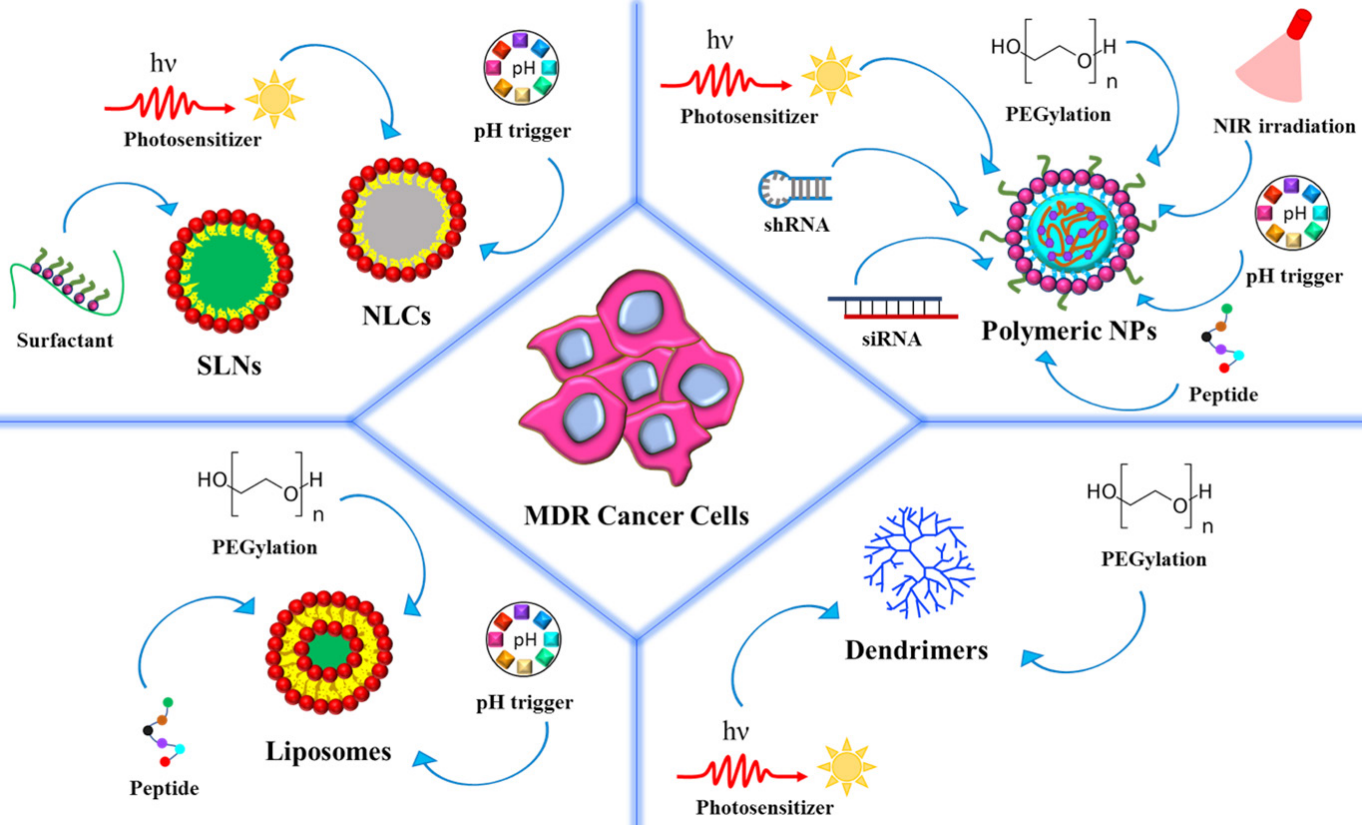
Scheme of the main organic nanoparticles investigated for counteracting MDR in cancer. From the left to the right side, liposomes, solid lipid nanoparticles (SLNs), nanostructured lipid carriers (NLCs), polymeric nanoparticles (NPs), and dendrimers are depicted. Possible functionalization with targeting ligands and external stimuli exploited for triggered drug release are reported. MDR: multidrug resistance

Organic nanoparticles present many advantages, among the other biocompatibility, due to their natural components (i.e., lipids, polymers) and biodegradability. They can be constituted by amphiphilic molecules that make them very versatile and, like inorganic nanocarriers, they can be precisely modified for targeted delivery. In the following sections, we will report some examples of the successes of these nanomaterials in counteracting MDR [Figure 3].

### Lipid-based nanoparticles

Liposomes are composed of a phospholipid bilayer and an aqueous core, therefore they are very versatile and can encapsulate both hydrophilic and hydrophobic molecules^[99]^. All these features make them minimally cytotoxic and highly biocompatible^[100]^. The possibility to encapsulate drugs into liposomes improves their stability, avoiding degradation during circulation^[101]^. They commonly accumulate in tumors thanks to the EPR effect, nevertheless, they are quickly eliminated by the reticuloendothelial system^[102]^. Therefore, PEGylation has been introduced for enhancing their stability and circulation times^[103]^. Thanks to surface functionalization, liposomes can be specifically and univocally targeted to tumor cells. They are usually internalized by endocytosis, thus avoiding MDR transporters^[104]^. Pegylated liposomes co-loaded with resveratrol and paclitaxel efficiently prompted cytotoxicity in MDR breast cancer cells and bioavailability and retention *in vivo*, inhibiting tumor growth in mice^[105]^. Interestingly, it has been demonstrated that some phospholipids can directly inhibit P-gp^[104]^ because they are direct substrates of this transporter^[106,107]^. Liposomes encapsulating carfilzomib and doxorubicin demonstrated highly efficient in tumor volume reduction and circumvention of MDR respect to the single drugs, both *in vitro* in multiple myeloma cells and *in vivo*^[108]^. It is important to mention that Vyxeos^TM^, constituted by liposomes loaded with cytarabine and daunorubicin, has been recently approved for the treatment of acute myeloid leukemia patients^[109]^. Notably, liposomes can be designed sensitive to external stimuli for getting targeted triggered release^[110]^. Jiang *et al.*^[111]^ fabricated liposomes modified with a peptide, pH-responsive, and with mitochondrial targeting properties, carrying paclitaxel to lung cancer cells A549 and A549 resistant to taxol and inducing apoptosis mediated by mitochondrial pathways. These nanomaterials efficiently inhibited tumor growth *in vivo*.

Solid lipid nanoparticles (SLNs) are constituted by lipids solid at body temperatures such as waxes, fatty acids, and glycerides and a stabilizing surfactant that can be added during fabrication^[112]^. They present higher drug stability and sustained release respect to liposomes, even though their crystallinity can induce low drug loading. A combination of encapsulated paclitaxel and verapamil showed drug release and MDR reversal in breast cancer cells^[113]^. Lipid nanoparticles loaded with doxorubicin and curcumin gave enhanced cytotoxicity and required reduced doses for obtaining MDR circumvention in hepatocellular carcinoma cells and in *in vivo* mice models^[114]^. The synthetic ether lipid edelfosine has demonstrated to possess antitumor properties in several cancers but, unfortunately, the leukemic cell line K-562 shows resistance to this compound. Using nanoencapsulation, cells underwent death by means of autophagy pathways and, after endocytosis, an increase in autophagic vesicles was appreciable, possibly overcoming MDR in this cell line^[115]^. SLNs have been also designed pH-responsive and, loaded with doxorubicin, released the molecule in the acidic microenvironment of MDR cells, limiting P-gp drug efflux^[116]^.

Although the many achievements, as mentioned above, some structural limits linked to SLNs crystallinity remain to be overcome. Therefore, nanostructured lipid carriers (NLCs) constituted by one or more liquid lipids have been recently introduced^[117]^. The partially crystalline solid matrices allow to pack higher amounts of payload, improving drug availability. These nanomaterials have been investigated as drug delivery systems for cancer therapy and MDR circumvention. NLCs were designed co-delivering paclitaxel and indocyanine green for combined chemo and photodynamic therapy. The nanoformulation stabilized and protected the drug, while laser irradiation favored drug release and ROS production, inducing increased cytotoxicity. Moreover, nanoparticles efficiently targeted tumors in mice, showing great promise for cancer therapy^[118]^. Nanostructured lipid carriers co-delivering β-lapachone, able to generate ROS, and the chemotherapeutic agent doxorubicin have been recently developed. Doxorubicin was efficiently delivered and accumulated in MCF-7/ADR breast cancer cells and in mice models of this tumor^[119]^. NLCs carrying doxorubicin and vincristine have been investigated for their efficacy in the treatment of human diffuse large B-cell lymphoma cell line and in a xenograft model, showing their ability to act against drug resistance^[120]^.

Lipid-based nanocarriers have demonstrated to be successful as drug delivery systems, however they present limited loading capacity and shelf life, can undergo oxidation, and modulation of drug release remains somehow difficult^[100]^. Therefore, polymeric carriers have been developed, with higher stability and the possibility to be easily fabricated with specific release features.

### Polymeric nanoparticles

Polymeric nanoparticles, thanks to their biocompatibility, have been widely explored as multifunctional nanomaterials for the delivery of drugs and macromolecules and also as potential tools for reversing MDR in cancer^[104]^. For instance, *in vitro* and *in vivo* researches demonstrated that taxol-loaded nanoparticles showed a toxicity higher than taxol itself in MDR human colon cancer cells, without displaying not specific cytotoxic effects^[121]^. Multifunctional polymeric nanoparticles carrying various drugs have been designed. For instance, nanocarriers loaded with paclitaxel and fluorouracil revealed more cytotoxic than each agent *per se* and efficiently reversed MDR^[122]^. Polymeric nanoparticles are extremely versatile and can be adapted to be responsive to external stimuli. For instance, pH-responsive nanocarriers based on acetylated α-cyclodextrin have shown improved efficacy of several anticancer agents in MDR tumors^[123]^. Notably, nanotools constituted by polymeric peptides forming a shell sensitive to tumor-specific enzymatic degradation have been designed^[124]^ and peptide chains sensitive to matrix metalloproteinases (MMPs) have been exploited for carrying anticancer agents^[125]^. In recent work, a copolymer (PEG2k-pp-PE) has been developed sensitive to MMP2 and able to inhibit P-gp, achieving MDR reversal^[126]^. Interestingly, lipid polymeric hybrid nanoparticles loaded with vincristine and quercetin were tested in MDR non-Hodgkin’s lymphoma cells, showing a sustained release profile and antitumor activity *in vivo*^[127]^.

These nanoparticles have been efficiently modified also for directly interfering with MDR mechanisms. Self-assembled nanoparticles, carrying doxorubicin and shRNA targeting the apoptosis-inhibiting gene survivin, demonstrated efficient in overcoming MDR in breast cancer cells^[128]^. Many systems have been designed using poly lactide-co-glycolide (PLGA), a biodegradable polymer, and surface modified for tumor targeting^[129,130]^. PLGA nanoparticles carrying paclitaxel and siRNAs directed against focal adhesion kinase efficiently overcome MDR, targeting CD44 positive ovarian cancer cells *in vitro* and xenografts^[131]^. Polymeric nanoparticles constituted of pH-sensitive PLGA-PEG-folate and cell-penetrating peptide R7-conjugated PLGA-PEG were constructed for delivering vincristine sulfate (VCR) overcoming MDR in breast cancer cells^[132]^. In an interesting study, docetaxel-loaded PLGA d-α-tocopheryl polyethylene glycol 1000 succinate (TPGS)/Poloxamer 235 nanoparticles were fabricated. Poloxamers are well-known co-polymers able to reverse MDR, interfering with P-gp efflux pumps. Nanoparticles were efficiently uptaken by cells in a MDR human breast cancer cell line and *in vivo*, showing great potential for therapeutic application^[133]^. PLGA nanoparticles, modified with d-α-tocopheryl polyethylene glycol 2000 succinate (TPGS2k) and loaded with SN-38, induced cytotoxicity in MDR A549 cells. Intriguingly, it was found that upon drug release, they were able to interfere with the structure and function of mitochondria, reducing ATP production, and consequently blocking P-gp efflux pumps^[134]^. Another molecule, podophyllotoxin, is known for its effects on P-gp activity, but unfortunately, it is poorly soluble and highly toxic, thus making it difficult its use in a clinical context. Therefore, upon conjugation with PEG and acetylated carboxymethyl cellulose (CMC-Ac), it selectively accumulated within tumors with improved efficacy in MDR cancers *in vivo*^[135]^.

Another category of organic nanoparticles based on polymeric macromolecules are DNA-based nanoparticles. They have been exploited as efficient platforms for binding intercalating agents used to treat cancer, displaying the advantage to easily self-assemble and to be precisely modifiable for incorporating targeting moieties. An interesting study was based on the fabrication of rod-like DNA origami nanocarriers able to overcome MDR in daunorubicin resistant leukemia cells. Thanks to their stable structure, the drug was efficiently delivered at higher doses and with better retention respect to the free drug, thus enhancing its efficacy. This study showed that DNA origami circumvented MDR efflux pumps giving great potential for its clinical application^[136]^.

### Polymeric micelles

These nanomaterials are composed of amphiphilic copolymers and present a hydrophilic shell and a hydrophobic core where drugs can be easily encapsulated^[137]^. Amphiphilic block copolymers constituted by Pluronic® are widely employed for encapsulating hydrophobic drugs^[138]^. Interestingly, it has been shown that Pluronic® can be internalized by endocytosis, localizes in mitochondria, and, by interfering with the respiratory chain, causes ATP depletion in MDR cells^[139]^. Some researches showed that Pluronic® P85 caused a reduction in ATP levels, specifically in MDR cells, correlating with decreased levels of P-gp^[140,141]^. Micelles composed by PEG, Pluronic® P123, and PEI blocks *via* disulfide bond and loaded with paclitaxel and siRNAs directed against polo-like kinase 1, effectively depleted ATP avoiding MDR^[142]^. Micelles constituted by diblock copolymers with a hydrophilic PEG block and a hydrophobic polyphosphoester block bearing a disulfide bond in a side group were administered to MDR cancer cells and doxorubicin was released. Upon disulfide bond break, micelles disassembled, reversing resistance. Similar results were obtained in mice xenografts, further corroborating their potential application in a clinical context^[143]^. A polymeric micelle has been designed able to increase size in acidic conditions and decrease it in the presence of high intracellular glutathione concentrations (typical of tumor cells). This system effectively delivered doxorubicin to the nucleus of MDR breast cancer cells^[144]^. An interesting theragnostic system was developed by Yu *et al.*^[145]^ consisting of pH- and NIR-responsive polymeric micelles carrying a doxorubicin prodrug. In acidic conditions (endosomes), the structure collapsed and, upon NIR irradiation (hyperthermia), penetration and release were enhanced in MDR breast cancer cells and *in vivo*. Micelles composed by a pH-responsive diblock copolymer, a polymeric prodrug of doxorubicin, and a photosensitizer were developed by the same group. The nanomaterial activated fluorescence signal in an acidic environment and, after NIR irradiation, ROS were induced and doxorubicin was released performing photodynamic therapy. NIR light was then converted into heat helping drug penetration, photothermal therapy, and photoacoustic imaging in MDR tumors^[146]^. Active targeting has been achieved by surface modification of pH-sensitive doxorubicin micelles with folic acid. Upon release of the drug from endosomes, due to low pH, the system was proved to overcome P-gp activity increasing drug accumulation and retention and finally overcoming MDR in human breast and ovarian cancer cells^[147-149]^.

### Dendrimers

Dendrimers are constituted by polymers composed of a dendritic skeleton arranged to form a spherical shape. They can deliver drugs attached to their surface or encapsulated in the formed cavity, offering improved stability and reduced cytotoxicity^[150]^. In an interesting work, a dendrimer carrying phthalocyanine and interacting with poly(ethylene glycol)-b-poly(*L*-lysine) block copolymer (PEG-PLL) constituting a complex with a micelle was combined with doxorubicin, endocytosed and accumulated in vesicular compartments upon irradiation. Doxorubicin was finally localized in the nucleus of MDR MCF-7 cells and in a xenograft model^[151]^.

Although polymer-based nanomaterials have proven to be highly functional in the altered tumor microenvironment, however, it has to be carefully considered that also normal cells possess similar mechanisms, therefore they can solicit potential undesired effects. Hence, precise targeting remains the essential requirement to be fulfilled before their clinical application.

## Conclusion

Nowadays, MDR is one of the biggest challenges to be faced by anticancer therapy and, up to now, only combination therapy has revealed effective as MDR clinical treatment even though with limited successes related to the extreme diversity distinctive of human tumors and the intrinsic properties of administered drugs. For instance, their different pharmacokinetic properties have led to several complications during *in vivo* translation. Nanomedicines can simultaneously encapsulate more than one molecule, can be functionalized for targeting, and promote controlled release, enhancing the effectiveness of combination therapy [Table 1]. Despite the several advantages respect to conventional treatments, currently, only seven clinical trials have been reported involving nanoparticles for the treatment of drug-resistant cancer patients. More in detail, five out of seven are based on the administration of paclitaxel albumin-stabilized nanoparticle formulations, alone or in combination with other compounds, in taxol resistant patients with metastatic breast cancer^[152]^, in platinum-resistant ovarian, fallopian tube, or primary peritoneal cancer^[153,154]^, in advanced gastric cancer^[155]^ and in platinum-resistant recurrent ovarian cancer^[156]^. These trials demonstrated the feasibility of the treatments in terms of tolerability and evidenced the success of the regimens in treated patients, placing the premises for their clinical approval. A formulation of camptothecin, conjugated to a hydrophilic, cyclodextrin-based linear polymer has been reported in a phase II clinical trial in subjects presenting recurrent platinum-resistant ovarian, tubal and peritoneal cancer, giving promising results in terms of safety, tolerability, and effectiveness^[157]^. Only one clinical trial, based on a polymeric nanoparticle (PNP) formulation containing the poorly soluble taxane docetaxel, administered to platinum-resistant ovarian cancer patients, is currently active and recruiting subjects^[158]^.

**Table 1:** Overview of the most relevant researches involving nanocarriers for overcoming multidrug resistance in cancer

Nanoparticle and drug	Targeting ligand	Cancer model	Mechanism	Results	Ref.
Iron oxide	siRNA P-gp	MDR human breast cancer cells Orthotopic mice	Silencing	P-gp downregulation *in vitro* and *in vivo*	[48]
Gold niosome Thymoquinone	siRNA Akt	MDR human breast cancer cells Xenograft mice	Silencing Activity of Thymoquinone	Akt downregulation *in vitro* Enhanced apoptosis *in vivo*	[56]
Gold Doxorubicin	Anti-DR-4 antibody	MDR human colorectal adenocarcinoma cells Xenograft mice	Activity of Doxorubicin	Reduced tumor growth *in vivo* upon NIR irradiation	[59]
CdSe/ZnSe QD	siRNA P-gp	MDR human cervical cancer cells	Silencing Activity of Doxorubicin	P-gp downregulation	[65]
Mesoporous silica Doxorubicin	siRNA P-gp	MDR human breast cancer cells Xenograft mice	Silencing Activity of Doxorubicin	P-gp downregulation *in vivo*	[78]
MWCNT	Anti-P-gp antibody	MDR human ovarian cancer cells	Antibody targeting	Strong phototoxicity in tumor spheroids	[91]
GO Adriamycin	siRNA miR-21	MDR human breast cancer cells	Silencing Activity of Adriamycin	Enhanced cytotoxicity *in vitro*	[93]
GO Doxorubicin	Molecular beacon MDR1 and ETS1	MDR human breast cancer cells Xenograft mice	Silencing Activity of Doxorubicin	MDR1 and ETS1 downregulation *in vitro* and *in vivo*	[97]
Liposome Paclitaxel	Mitochondrial targeting peptide	MDR human lung cancer cells Xenograft mice	Activity of Paclitaxel	Enhanced apoptosis *in vitro* Reduced tumor growth *in vivo*	[111]
Polymeric Doxorubicin	shRNA Survivin	MDR human breast cancer cells Tumor bearing mice	Silencing Activity of Doxorubicin	Survivin downregulation P-gp and GST inhibition *in vitro* Antitumor activity *in vivo*	[128]
Polymeric Paclitaxel	siRNA FAK	MDR human ovarian cancer cells Xenograft mice	Silencing Activity of Paclitaxel	Cytotoxicity and apoptosis *in vitro* Reduced tumor growth *in vivo*	[131]
Polymeric Vincristine sulfate	R7 peptide Folate	MDR human breast cancer cells Tumor bearing mice	Activity of Vincristine sulfate	Enhanced cytotoxicity *in vitro* Antitumor activity *in vivo*	[132]
Polymeric micelle Paclitaxel	siRNA PLK1	MDR human colorectal adenocarcinoma cells Tumor bearing mice	Silencing Activity of Paclitaxel	ATP depletion and PLK1 inhibition *in vitro* Reduced tumor growth *in vivo*	[142]
Polymeric micelle Doxorubicin	Folate	MDR human breast cancer cells Xenograft mice	Activity of Doxorubicin	Enhanced cytotoxicity *in vitro* Reduced tumor growth *in vivo*	[147]
Polymeric micelle Doxorubicin	Folate	MDR human ovarian cancer cells Xenograft mice	Activity of Doxorubicin	Enhanced cytotoxicity *in vitro* Reduced tumor growth *in vivo*	[149]

MDR: multidrug resistance; P-gp: P-glycoprotein; QD: quantum dot; GO: graphene oxide

It is evident that some challenges remain to be tackled before expanding nanoparticles application in clinics. Indeed, it has to be considered that, once delivered to their target tumor, drugs can behave unexpectedly depending on the complexity of the entire organism^[159]^. It is also important to mention that nanomaterials can be sequestered and retained by healthy organs, thus raising safety concerns and potentially inducing resistance in the long-term period^[160]^. It is of foremost importance to investigate the fate of nanotherapeutics in terms of tissue accumulation and safety characterization: nanomaterials can perform differently in living organisms and can provide toxicity due to their peculiar surface chemistry^[161]^. Some studies reported damages to cell membranes, organelles, and DNA^[162]^ and immune responses induction^[163]^. Much effort needs to be gathered from different disciplines for answering several open questions and for developing *in vitro* models to assess nanoparticles toxicity (rapid, inexpensive), *ex vivo* models to define administration routes, and *in vivo* models to establish absorption, distribution, metabolism, and excretion. Last but not least, large-scale production of nanocarriers with precise drug ratios continues to be difficult and further improvements are required before application in the clinical context. The definition of *ad hoc* regulations remains an essential issue to guarantee suitable measures for nanomedicine clinical translation^[164,165]^.

## Future perspectives

Nanomedicine and the development of nanocarriers responsive to precise stimuli have allowed great advances in combating MDR. However, some issues remain to be faced due to the extreme heterogeneity of the tumor environment. The mechanisms responsible for MDR are also very complicated and may vary between tumor cells and individuals. Moreover, it has to be considered that, although not targeted nanocarriers can accumulate exploiting the EPR effect, main researches are performed on animal models that do not perfectly mimic tumor growth in the human body. It is therefore critical to develop active targeting nanomedicines, characterized by precise drug delivery and release. Further studies are required for investigating nanomaterials biocompatibility and safety, together with additional researches concerning MDR pathways. In the next future, personalized nanotherapies for counteracting MDR in cancer patients could be envisaged.

## Declarations

### Copyright

© The Author(s) 2020.
